# Alternating Biplanar Fluoroscopy in Percutaneous Nephrostomy to Approach Stones in Patients With Horseshoe Kidney: An Institutional Experience

**DOI:** 10.7759/cureus.16542

**Published:** 2021-07-21

**Authors:** Ahmed Al Muzrakchi, Loai J. A. Aker, Ali Barah, Ala Alsherbini, Ahmed Omar

**Affiliations:** 1 Radiology, Hamad Medical Corporation, Doha, QAT; 2 Imaging Department, Royal Melbourne Hospital (Melbourne Health), Melbourne, AUS

**Keywords:** percutaneous nephrostomy, alternating biplanar, fluoroscopy, horseshoe kidneys, kidney stones

## Abstract

This case series aims to evaluate the use of alternating perpendicular biplanar fluoroscopy in percutaneous nephrostomies/percutaneous nephrolithotripsies (PCNs/PCNLs) to approach renal stones in patients with horseshoe kidneys. Between January 2012 and December 2019, PCNs/PCNLs were done for six patients with horseshoe kidneys having renal stones. Skin and renal calyceal entry points were determined by alternating perpendicular biplanar fluoroscopy using a portable C-arm machine in the conventional fluoroscopy unit. The site of renal access, postoperative complications, and residual stones was assessed.

The mean age of the patients was 36.8 years. The mean stone size was 6.1 cm (2.1-16.05cm). In five out of six (5/6; 83%) patients, there was one access site. Four patients had their access site through the upper calyx, and one patient had it through the lower calyx. The stone-free rate was four out of six (4/6; 66.6%). One patient had a mild drop in hemoglobin postoperatively. There were no major complications reported.

The implementation of alternating biplanar fluoroscopy was found safe and helpful in providing a better appreciation of renal anatomy and stone location in patients with horseshoe kidneys. This technique helps in approaching horseshoe kidney stones in PCN/PCNL without moving the patient or fluoroscopy machine, with a potential decrease in operation time and radiation exposure.

## Introduction

The horseshoe kidney represents a congenital renal fusion anomaly with an abnormal position and rotation of the horseshoe kidneys that complicate the management of stones in these kidneys. The current treatment modalities include shock wave lithotripsy (SWL), retrograde intrarenal surgery, and percutaneous nephrolithotripsy (PCNL) [1]. PCNL is indicated for the management of large (> 2 cm) stones or in failed SWL [2].

In this case series, we report our eight-year institutional experience in utilizing alternating biplanar fluoroscopy in percutaneous nephrostomies/percutaneous nephrolithotripsy (PCN/PCNL) for approaching renal stones in patients with horseshoe kidneys. With this technique's success and convenience, we recommend its implementation not only for horseshoe kidney stones but also as a simple technique for approaching normal obstructed kidneys.

## Case presentation

This case series was based on six patients who had horseshoe kidney stones, which were diagnosed in the emergency department or outpatient clinic and were referred to the interventional radiology suite in our referral center for PCN/PCNL between January 2012 to December 2019. Data obtained from the electronic system included age, gender, radiologic investigations and procedure, stone-related data (size of kidney, collecting system dilatation, stone number, and location), access site, and postprocedure complications (Table 1).

**Table 1:** The individual characteristics of patients with stones in horseshoe kidneys treated with PCN/PCNL utilizing alternating biplanar fluoroscopy PCN: percutaneous nephrostomy; PCNL: percutaneous nephrolithotripsy

	Patient 1	Patient 2	Patient 3	Patient 4	Patient 5	Patient 6
Age	32	34	37	55	27	36
Gender	male	male	male	male	male	male
Stone size	2.1 cm	6.05 cm	16.04 cm	4.9 cm	2.9 cm	4.92 cm
Stone location	Multiple stones in the pelvis, upper, mid, and lower calyces of the left kidney	Multiple stones in the isthmus and lower calyces of the right kidney	multiple stone aggregates in the pelvis, upper, mid, and lower calyces of the right kidney	multiple stones in the pelvis and upper minor calyces of the right kidney	multiple stones in the pelvis and lower collecting system of the left kidney	multiple stones in the isthmus and pelvis and lower calyces of the right kidney
Number of accesses	1	1	2	1	1	1
Access place	The lower minor calyx	The upper minor calyx	The upper and lower minor calyces	The right upper minor calyx	The upper minor calyx	The upper minor calyx
Operation time/ min (PCN+PCNL)	23+103 = 126	35+162 = 197	32+83 = 105	33+120 = 155	30+120 = 180	37+110 = 147
Residual stone	Yes, one stone	Yes, multiple fragments	No	No	No	No stone postoperatively; however, had a new 8 mm stone after 4 months
Residual stone size	2 mm	23.6 mm	N/A	N/A	N/A	8 mm
complications	Hemoglobin drop by one unit	None	None	None	None	None
Indication of procedure	Urosepsis with renal stone, and failed SWL	Large stone with failed SWL.	Large stones	Large stones	Large stones	failed SWL

Inclusion criteria: patients with horseshoe kidney presenting with symptoms of obstructive uropathy and/or infection and found in a radiologic exam to have a stone > 2 cm or underwent SWL treatment and failed. Six patients who met the inclusion criteria underwent PCN/PCNL. The procedures involved a portable C-arm machine to provide lateral fluoroscopy whilst the conventional fluoroscopy unit provided the anteroposterior (AP) view, resulting in alternating perpendicular image planes.

While the patient was in the prone position, local anesthesia was used with mild general sedation. Nephrolithotomy was applied under general anesthesia. The collecting system was visualized by direct puncture with an 18-gauge trocar or Chiba needle and then a wire was passed into the collecting system. Then, the needle was removed, and a dilator was placed over the wire. Afterward, the contrast was injected into the collecting system.

The calyceal and skin entry points were determined based on the stone location, and configuration of the collecting system. The calyceal entry point (CEP) is usually the posterior lower minor calyx in cases of posterior nephrostomy. In nephrolithiasis, entry through the posterior minor calyx is preferred when there is a staghorn calculus, a renal pelvis stone, a stone in the posterior lower minor calyx, or a stone in the anterior or posterior upper calyx. However, If the stone lies in the posterior or anterior middle minor calyx or anterior lower minor calyx, the entry is preferred to be through the calyx containing the stone.

The fluoroscopy was first done in the lateral view (using the C-arm), showing contrast-filled minor calyces lying in dorsal and ventral rows. The patient was then fluoroscoped in the AP view. The minor calyces would lie in lateral and medial rows representing the anterior and posterior minor calyces, respectively. The posterior lower minor calyx is the most caudal calyx in the dorsal row in the lateral view and the medial calyx of the lower calyceal group in the anteroposterior view.

**Figure 1:**
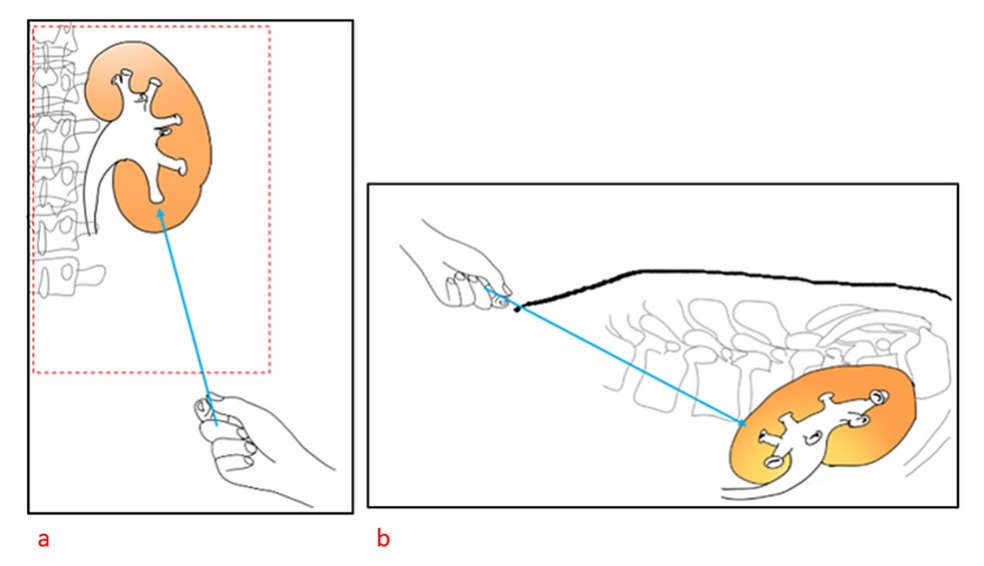
This shows the correct proposed nephrostomy tract (PNT) (blue arrow) in both the anteroposterior (a) and lateral views (b)

Then, the skin entry point was identified. On lateral fluoroscopy, a sterile metallic pointer was maneuvered to lie just dorsal to the calyceal entry point. On AP fluoroscopy, the pointer was moved caudally until the line of the proposed nephrostomy track is directed toward the longitudinal axis of the lower major calyx. This point should be below the 12th rib and just lateral to the outer border of the paraspinal muscle. The skin entry point was punctured with an 18-gauge needle, which was advanced 2-3 cm into the subcutaneous tissue, then gradually advanced under alternating perpendicular biplanar fluoroscopy guidance, with the following main considerations:

A: The proposed nephrostomy tract (PNT) should be directed towards the CEP at all times as seen in AP and lateral views in fluoroscopy until puncturing the CEP (Figure 1).

**Figure 2:**
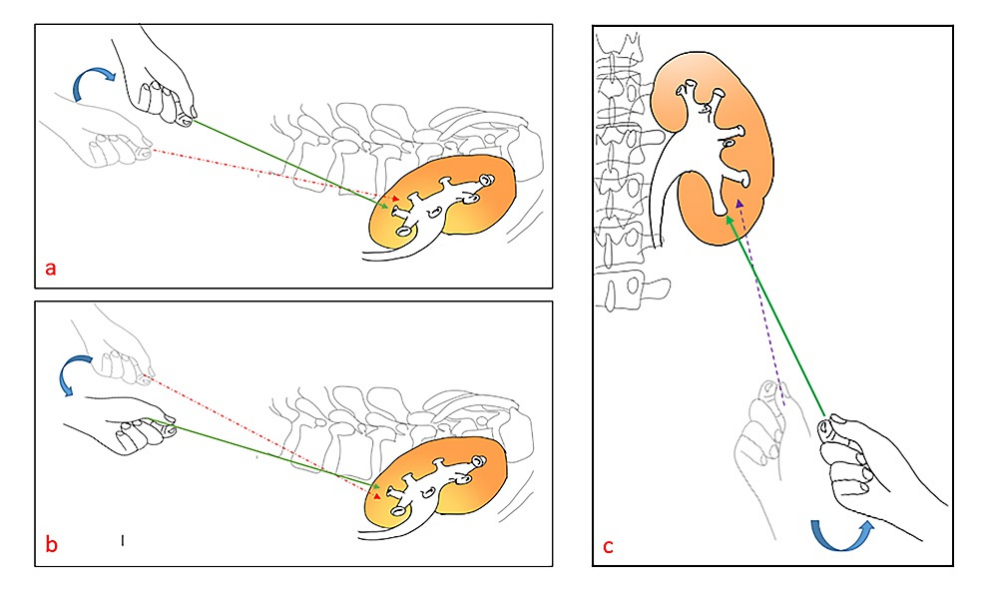
In the lateral view (a), the incorrect orientation of the proposed nephrostomy tract PNT (dashed red arrow) is directed dorsally to the tract of the lower calyx. So, the operator's hand should be tilted cranially to adjust the PNT correctly (solid green arrow). The incorrect orientation of the PNT (dashed red arrow) in the lateral view (b) is directed caudally to the tract of the lower calyx. Therefore, the operator's hand should be tilted caudally to adjust the needle to the right tract (solid green arrow). (c) shows the incorrect orientation of the PNT (dashed purple arrow) as it is directed lateral to the tract of lower calyx in anteroposterior view. Therefore, the operator's hand should be tilted laterally (ventrally) to adjust the needle to the right tract (solid green arrow)

B: First, using the lateral view, if the PNT seems to be dorsal to the CEP, the operator should correct the needle direction by tilting the proximal end of the needle cranially until the needle direction is pointing towards the CEP. The opposite should be done if the needle track is directed caudally to the CEP on the lateral view (Figure 2, panels a-b). The operator should fix the position of the needle in the correct position, then proceed to Step C.

C: In the AP view, if the PNT direction is lateral to CEP, the proximal end of the needle should be tilted ventrally (laterally) to recorrect the needle tract (Figure 2, panel c).

The needle was then advanced 1-2 cm deeper, with the same observations done in steps A-C until the needle tip was seen projecting on the target in both AP and lateral views. Additional technical details were previously reviewed [3-4].

Once the calyx is punctured, the stylet of the needle should be withdrawn and followed by a free backflow of urine. The guidewire was then advanced through the cannula into the minor calyx, the major calyx, and on into the pelvis of the kidney. The catheter was then inserted over the guidewire in the case of nephrostomy. But in nephrolithotomy, the tract was dilated by telescopic metallic dilators, and a metallic sheath was inserted. Small stones (1.2 cm or less on radiography) were extracted whole under biplanar fluoroscopy while larger stones were removed in fragments under direct vision using a nephroscope or by a laser device.

The same technique was applied for stones in horseshoe kidneys with minimal modifications needed because the renal congenital anomaly and the location of the stone and the needle can be visualized in both perpendicular planes alternatively.

Results

**Figure 3:**
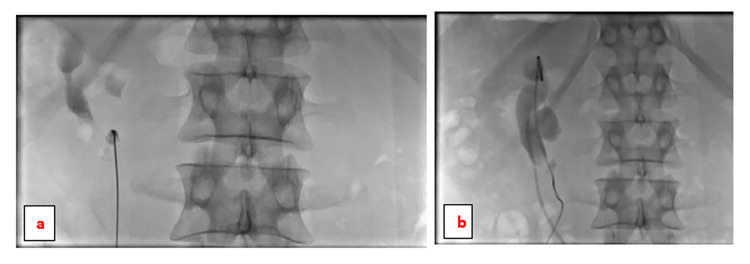
Fluoroscopic guidance of entering the horseshoe kidney through the inferior calyx in the anteroposterior view (a & b) The guidewire was advanced through the inferior calyx up to the renal pelvis and upper calyx.

The mean age of the patients was 36.8 years (range 27-55 years). All patients were male. Four patients had a stone on the right side, and two patients had left-sided stones. Three patients underwent SWL previously which failed to clear the stone completely. two had a history of prior renal surgery. Most of the patients presented with a history of flank pain. One patient presented with a renal stone, and symptoms/signs of urosepsis. The individual characteristics of the patients are shown in Table 1. Stone size was measured by summing the diameter of the multiple stones found in a renal unit. The mean stone size was 6.1 cm (2.1-16.05 cm). In 5/6 (83%) patients, there was one access site. Four patients had their access site through the upper calyx, and one patient had it through the lower calyx. One patient (17%) had two access sites; through the upper and lower calyces. Figure 3 demonstrates the fluoroscopic guidance of accessing a horseshoe kidney through the lower calyx.

**Figure 4:**
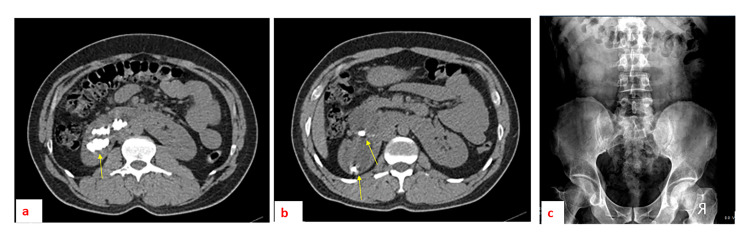
A patient with selected axial sections of plain abdominal CT demonstrating multiple renal stone aggregates (a and b) located in the upper and lower calyceal groups in the right kidney before the percutaneous nephrostomy/nephrolithotomy. An abdominal X-ray one month after the procedure (c), with no residual stones seen in the kidney areas.

The stone-free rate was found to be 4/6 in our study (66.6%) (Figure 4).

No major complications were reported in the patients. One patient had mild hemoglobin drop but did not require transfusion. All patients had their nephrostomy tube removed within 48 hours postoperatively and discharged home, except one patient who had urosepsis preoperatively that required a longer hospitalized treatment. Patients were followed with abdominal X-rays for six months. One of the patients had a stone recurrence on X-ray. However, none of the patients, even those with residual stones, became symptomatic during follow-up.

## Discussion

With newer advancements in interventional radiology and urology, different minimally invasive treatment options became available to address stones in horseshoe kidneys. These options include SWL, PCN/PCNL, ureteroscopy, and retrograde intrarenal surgery. SWL treatment was recommended for stones less than 2 cm in the horseshoe kidney. On the other hand, a percutaneous approach to tackle the stone in the horseshoe kidney is advised if SWL fails or for bigger stones [3]. By reviewing the horseshoe kidneys' PCN/PCNL results in some previous studies, the success rate was considered if all stones are removed or if the residual stone was less than 4 mm, which collectively ranged between 50%-87.5% (Table 2) [4-9].

**Table 2:** The results of a few studies about percutaneous nephrostomy/nephrolithotomy as a treatment method for stones in horseshoe kidney patients PCN+PCNL: percutaneous nephrostomy + percutaneous nephrolithotomy; SWL: shock wave lithotripsy

Study group	Number of patients	Average stone size	Stone free rate	Complications	Comments
Suelozgen et al. [4]. 2015	6	1007 mm2 (375-1480),	50%,	Hematocrit drop in 66%. None needed a transfusion.	The average operation time was 117.1 min. (44-250 min)
Al-Otaibi et al. [9]. 1999	12	N/A	75%	N/A	More than one nephrostomy tract was needed in 5 patients.
Satav et al. [7].	23	22.03 ± 10.33 mm	87.50%	Postoperatively, two patients had a fever, which was treated conservatively	The mean operation time was 67.22 ± 7.63 minutes. One patient with a staghorn stone needed open surgery.
El Ghoneimy et al. [6].	21 renal units (17 patients	N/A	85.7%	Intraoperative calyceal neck injury in one case, causing minor bleeding. Major renal pelvis perforation in one case, and one case of postoperative urosepsis.	The operative time ranged from 30 min to 160 min (mean 70 min).
Etemedian et al. [5].	21	37.2 ± 16.6 mm	71.40%.	Minor complications were reported in 3 (14.28%) patients; transfusion in one (4.76%), fever in one (4.76%), and ileus in one (4.76%) patient	
Darabi Mahboub et al. [8].	9	N/A	66.7%	Access secured in 8/9 patients. One patient needed open renal surgery	Two patients underwent SWL for residual stones.

Our study shows a comparable stone-free rate of 66.6% with a desirable profile of minimal complications. 

In horseshoe kidneys, the ureter usually originates from the upper part of the pelvis, which increases the risk of ureteropelvic junction obstruction and difficult calculus passage. Accessing the kidney through the upper pole not only facilitates access to the upper pole calyces but helps in accessing the pelvis, the calyces of the lower pole, and the proximal ureter [8]. Most of the patients in our study had puncturing through the upper calyx, and all were successful from the first attempt. One patient had access through the lower calyx, and one had upper and lower calyces puncturing.

The use of fixed alternating perpendicular biplanar fluoroscopy for entering the kidney provided a helpful appreciation of abnormal renal anatomy and obstructing stones while obviating the need of tilting the patient or fluoroscopy machine. This might lead to less potential operation time and radiation exposure to both the operator and the patient. Also, the technique would be easier to learn for interventionists in training. However, due to the small patient population and paucity of related studies in the literature, further evidence-based recommendations need more investigation.

Common complications related to the PCNL procedure include fever, bleeding, urine leakage, and symptoms caused by residual stones [4]. In our study, one patient had a minimal drop in hemoglobin but did not require any transfusion. Vascular injury risk during horseshoe kidney access is not higher than normal kidney access since the vessels of the horseshoe kidney enter the hilum from the anteromedial site with the posterior rotation of the calyxes [4,10]. Complications like a renal or gastrointestinal injury can occur after PCN/PCNL [4,6,11]. Yet, no major complications occurred in our study.

We utilized two perpendicular planes of fluoroscopy imaging with images being displayed on two separate monitors. This allowed entering the skin below the level of pleural reflection and provided three-dimensional monitoring for the advancement of the puncturing needle toward the target entry point. Therefore, the risk of pneumothorax becomes less likely to occur [12].

There is less definite evidence regarding the algorithm of management of stones in the anomalous kidney in comparison to the normal kidney. Less invasive treatment options like extracorporeal shock wave lithotripsy (SWL), and ureteroscopy (URS) were found safe and effective in horseshoe kidney patients with a stone <2 cm [13]. On the other hand, multiple studies found that PCL was considered safe and effective in removing calculi larger than 2 cm in horseshoe kidneys [8,14]. Yet, further recommendations and comparison with other treatment modalities require further research and evaluation.

The case series was limited by the small heterogeneous patient population and limited follow-up time. Due to the paucity of related studies in the literature, further evidence-based recommendations need more investigation.

## Conclusions

The use of alternating perpendicular biplanar fluoroscopy provided a better appreciation of the abnormal anatomy and stone localization in horseshoe kidneys in PCN/PCNL. Our case series shows that the implementation of biplanar fluoroscopy in PCN/PCNL in horseshoe kidney stones provided a stone-free rate of 66.6% that is comparable with other similar studies in the literature and with a desirable profile of minimal complications. The success and safety of this technique also make it suitable for use in normal kidneys with obstructing stones.
